# Optimized integration of mechanical circulatory support and endovascular therapy in pulmonary embolism-induced cardiac arrest: A case series

**DOI:** 10.1097/MD.0000000000046531

**Published:** 2025-12-12

**Authors:** Yuanjun Zhang, Zheng Lei, Ji Luo, Dongju Chen, Haojie Xiong, Li Zhang, Bo Wang, Hao Yang

**Affiliations:** a Department of Critical Care Medicine, West China Hospital of Sichuan University-Ziyang Hospital, Ziyang Central Hospital, Ziyang, Sichuan, China; b Department of Critical Care Medicine, West China Hospital, Sichuan University, Chengdu, Sichuan, China.

**Keywords:** cardiac arrest, case report, endovascular therapy, mechanical circulatory support, pulmonary embolism

## Abstract

**Rationale:**

Pulmonary embolism-induced cardiac arrest (PE-CA) remains a life-threatening condition with high mortality despite conventional management strategies. This case series explores the potential benefits of combining mechanical circulatory support (MCS) and endovascular therapy (ET) for optimizing outcomes in PE-CA.

**Patient concerns:**

Seven patients with PE-CA, presenting with sudden respiratory distress, hypotension, and loss of consciousness, were treated at our institution between January 2021 and May 2025. These patients exhibited significant hemodynamic instability, requiring immediate intervention to restore circulatory support and address the underlying thrombotic burden.

**Diagnoses:**

Diagnosis was confirmed via computed tomography pulmonary angiography and echocardiography, revealing extensive pulmonary emboli and evidence of right ventricular strain.

**Interventions:**

Patients were categorized into 3 treatment groups: conventional therapy (n = 2), single intervention (either MCS or ET, n = 2), and combined therapy (MCS + ET, n = 3). The combined therapy group received early MCS to restore circulatory support, followed by ET to remove the thrombotic burden.

**Outcomes:**

Descriptive analysis suggested that combined therapy was associated with shorter return of spontaneous circulation times (12–16 minutes vs 20–32 minutes in other groups) and favorable neurological recovery in all 3 cases. However, no statistical comparisons were performed due to the small sample size and heterogeneity of the cohort.

**Lessons:**

This case series highlights the feasibility of integrating MCS and ET in PE-CA management. While the findings suggest potential synergistic interactions between these therapies, larger comparative studies are required to confirm these preliminary observations and establish evidence-based protocols.

## 1. Introduction

Pulmonary embolism (PE) remains a significant threat to life and a major cause of nontraumatic mortality globally. According to epidemiological studies, the annual incidence of PE is approximately 100 to 200 cases per 100,000 population, with 2% to 5% of these cases progressing to fatal pulmonary embolism-induced cardiac arrest (PE-CA),^[1,2]^ this condition is associated with an alarmingly high mortality rate of 65% to 95%, even when managed under optimal medical conditions.^[3]^

The pathophysiological mechanism underlying PE-CA is primarily characterized by acute right ventricular dysfunction resulting from a substantial thrombotic burden within the pulmonary arteries. Also, the resultant right ventricular dilation shifts the interventricular septum leftward, further compromising left ventricular filling, reducing cardiac output, and decreasing systemic blood pressure.^[4]^ Additionally, acute right ventricular dilation may also induce right heart ischemia and myocardial injury, further deteriorating cardiac contractile function and ultimately leading to pulseless electrical activity (PEA) or ventricular fibrillation, culminating in cardiac arrest (CA).^[5]^ This dual “anatomical-functional” imbalance makes PE-CA exceptionally challenging to manage.

Conventional management strategies for PE-CA include standard cardiopulmonary resuscitation (CPR), systemic thrombolysis, and emergency surgical thrombectomy, each with some limitations. Standard CPR has limited effectiveness in patients with severe right heart failure, often failing to generate sufficient perfusion pressure to sustain critical organ function.^[6]^ While systemic thrombolysis can be rapidly administered, it carries a high risk of bleeding, and its thrombolytic effect may not develop quickly enough to rescue patients with severe hemodynamic instability.^[7]^ Emergency surgical thrombectomy, although effective for appropriate candidates, requires specialized surgical teams and often cannot be tolerated by patients in CA due to the procedural waiting time.^[8]^

Recent advancements in mechanical circulatory support (MCS) and endovascular therapy (ET) technologies have introduced new potential in the management of PE-CA. MCS devices, particularly extracorporeal membrane oxygenation (ECMO) and percutaneous cardiopulmonary support, can rapidly establish artificial circulation, restore perfusion to critical organs, and provide valuable time windows for intervention in patients.^[9]^ Some research has shown that in PE-CA patients who are unresponsive to conventional CPR, extracorporeal cardiopulmonary resuscitation (ECPR) can increase survival rates from <10% to 30–40%.^[10]^ Additionally, catheter-directed endovascular therapies enable direct and rapid removal of pulmonary arterial thrombi, thereby promptly reducing pulmonary vascular resistance and right heart burden.^[11]^ Furthermore, a multicenter registry study involving 287 patients with massive PE demonstrated a 96.5% technical success rate for ET, with a 30-day all-cause mortality of only 12%, which is significantly lower than historical data.^[12]^

Despite the promising individual contributions of MCS and ET to PE-CA management, systematic research on the optimal integration of these technologies in critically ill patients remains limited. Theoretically, MCS can provide stable hemodynamic support, creating a procedural time window and safety margin for ET, while ET can rapidly remove thrombi, allowing patients under MCS support to become less dependent more quickly.^[13]^ This case series aims to illustrate the potential benefits of integrating MCS and ET in managing PE-CA through detailed presentation of 7 representative cases treated with different strategies at our institution.

## 2. Case description

### 2.1. Case 1: conventional therapy

A 62-year-old male with history of recent knee replacement surgery 2 weeks prior (The Wells Clinical Prediction Rule for Pulmonary Embolism [Wells score] 6, high PE probability) presented to the emergency department with sudden-onset severe dyspnea and chest pain. While in the triage area, the patient developed CA with PEA. The patient received high-quality CPR, and endotracheal intubation was performed during the third cycle of CPR with minimal interruption of chest compressions, multiple doses of epinephrine were administered. Bedside echocardiogram during CPR showed severely dilated right ventricle (right ventricle [RV]/left ventricle [LV] ratio > 1.5), moderate tricuspid regurgitation with estimated systolic pulmonary artery pressure of 65 mm Hg, and leftward bowing of the interventricular septum, raising high suspicion for PE.

Given the high clinical suspicion for PE, alteplase 100 mg was administered intravenously (10 mg bolus followed by 90 mg over 2 hours) at 15 minutes into resuscitation efforts. Laboratory results later revealed markedly elevated D-dimer levels (8.7 μg/mL, normal < 0.5 μg/mL).

ROSC was achieved after 28 minutes of resuscitation. Post-ROSC CT pulmonary angiography confirmed bilateral large proximal pulmonary artery thrombi with nearly complete occlusion of the right main pulmonary artery. Post-resuscitation care included therapeutic anticoagulation with unfractionated heparin (target aPTT 60–80 seconds), vasopressor support (norepinephrine 0.5 μg/kg/min, vasopressin 0.04 units/min), and lung-protective ventilation strategies.

Despite initial stabilization, the patient developed progressive multiorgan failure. Neurological assessment revealed absent pupillary and corneal reflexes at 72 hours post-arrest. The patient eventually died on day 14 of hospitalization with significant hypoxic brain injury as the primary cause of death.

### 2.2. Case 2: conventional therapy

A 71-year-old female with metastatic breast cancer (Wells score 7.5, high PE probability) was admitted with progressive lower extremity swelling and worsening shortness of breath over several days. During routine vital sign monitoring on hospital day 1, she suffered CA with PEA rhythm.

CPR was immediately initiated per protocol, a point-of-care ultrasound revealed acute right ventricular strain with RV/LV ratio > 1.2 and paradoxical septal motion. Given her high-risk profile for PE, she received systemic thrombolysis with urokinase (3 million units intravenously) after 12 minutes of CPR. Laboratory values later confirmed elevated D-dimer (10.2 μg/mL) and troponin I (0.8 ng/mL).

ROSC was achieved after 32 minutes of resuscitation efforts. Subsequent computed tomography pulmonary angiography (CTPA) confirmed multiple segmental and subsegmental pulmonary emboli involving all lobes with evidence of right heart strain. Post-resuscitation management included mechanical ventilation, high-dose vasopressors (norepinephrine 0.8 μg/kg/min, epinephrine 0.1 μg/kg/min), and therapeutic anticoagulation with enoxaparin (1 mg/kg twice daily).

The patient remained hemodynamically unstable requiring escalating vasopressor support, persistent hypoxemia despite maximal ventilatory support (fraction of inspired oxygen [FiO_2_] 1.0, positive end-expiratory pressure 14 cm H_2_O). Serial neurological examinations demonstrated absent cortical responses and minimal brainstem reflexes. She survived the initial resuscitation but exhibited poor neurological recovery (cerebral performance category [CPC] 4) and died 18 days after admission due to progressive respiratory failure and multiorgan dysfunction.

### 2.3. Case 3: single intervention (MCS)

A 58-year-old male with history of unprovoked deep vein thrombosis 5 years prior (discontinued anticoagulation 2 years ago) presented to the emergency department with syncope and respiratory distress, shortly after arrival, he developed CA with PEA. After 10 minutes of conventional CPR without ROSC and with clinical suspicion of massive PE (Wells score 9, high PE probability), the decision was made to implement veno-arterial ECMO support.

The ECMO team was rapidly mobilized, and cannulation was performed under ongoing CPR with ultrasound guidance. ECMO flow was established at 4.2 L/min with sweep gas flow of 4 L/min and FiO_2_ 1.0. ECMO was successfully established, achieving artificial circulation at 22 minutes from arrest onset. Initial ECMO circuit parameters showed venous saturation of 38%, indicating severe tissue hypoxia.

Post-ECMO stabilization allowed for CTPA, which confirmed massive bilateral pulmonary emboli with CT obstruction index of 32/40. Laboratory values demonstrated severely elevated D-dimer (15.6 μg/mL), lactate (12.3 mmol/L), and evidence of right ventricular strain on biomarkers (N-terminal pro-B-type natriuretic peptide 12,500 pg/mL, troponin I 1.2 ng/mL). The patient was managed with therapeutic anticoagulation (unfractionated heparin, target anti-Xa level 0.3–0.7 IU/mL).

He required 7 days of ECMO support with slow weaning of flow rates as native cardiac output improved. Distal perfusion catheter was placed in the right superficial femoral artery to prevent limb ischemia. He was successfully decannulated on day 7 as right ventricular function recovered to near-normal (tricuspid annular plane systolic excursion (TAPSE) improved from 8–17 mm).

He survived to hospital discharge with moderate neurological disability (CPC 2) including short-term memory deficits and mild cognitive impairment, and continued to improve during outpatient rehabilitation.

### 2.4. Case 4: single intervention (ET)

A 49-year-old female who was 6 weeks post-cesarean delivery presented with acute onset of dyspnea and presyncope. Wells score was 8.5, indicating high PE probability. Shortly after arrival, she suffered CA with PEA. Standard CPR was initiated immediately with endotracheal intubation secured during the second cycle of compressions.

Return of spontaneous circulation (ROSC) was achieved after 18 minutes of resuscitation efforts with multiple doses of epinephrine (total 4 doses). Post-ROSC, she remained severely hypotensive requiring high-dose vasopressors (norepinephrine 1.2 μg/kg/min, vasopressin 0.04 units/min). Emergent bedside echocardiogram demonstrated severe right ventricular dysfunction (TAPSE 7 mm), severe tricuspid regurgitation (estimated RVSP 68 mm Hg), and McConnell sign (akinesia of the RV free wall with normal apical contractility), pathognomonic for acute pulmonary embolism (PE). CTPA confirmed saddle pulmonary embolus with extensive bilateral thrombus burden (CT obstruction index 28/40). Laboratory studies showed markedly elevated D-dimer (18.2 μg/mL) and troponin I (1.8 ng/mL).

She underwent successful catheter-directed thrombus aspiration with the AngioVac system, achieving significant reduction in thrombus burden. Pulmonary angiogram before and after intervention demonstrated improvement in pulmonary blood flow with reduction in pulmonary arterial pressure from 65/32 mm Hg to 42/18 mm Hg.

Despite the technical success, she experienced several episodes of recurrent arrest during the procedure, requiring intermittent CPR with a total additional ROSC time of 24 minutes. Throughout the procedure, she required escalating doses of vasopressors and inotropes (epinephrine infusion 0.2 μg/kg/min, milrinone 0.5 μg/kg/min) to maintain circulation while the thrombus was being removed.

Post-procedure, she required prolonged intensive care with 9 days of mechanical ventilation and gradually decreasing vasopressor support. Serial echocardiograms showed progressive improvement in right ventricular function (TAPSE improved to 15 mm by day 5, and 19 mm by discharge). She ultimately survived with good neurological outcomes (CPC 1) and was discharged to rehabilitation on day 24.

### 2.5. Case 5: combined therapy (MCS + ET)

A 52-year-old male with recent history of pelvic fracture and prolonged immobilization (Wells score 7, high PE probability) presented with sudden cardiopulmonary collapse at home. Upon arrival of emergency medical services, he was in CA with PEA. Prehospital ACLS was initiated with high-quality CPR and endotracheal intubation.

Upon arrival at the emergency department with ongoing CPR, point-of-care echocardiography revealed severely dilated right ventricle (RV/LV ratio > 1.8), flattened septum, and minimal LV filling consistent with acute right heart strain. Laboratory studies drawn during resuscitation later revealed markedly elevated D-dimer (16.8 μg/mL), troponin I (2.1 ng/mL), and lactate (14.6 mmol/L).

The ECMO team was mobilized simultaneously with ongoing resuscitation efforts. ECMO flow was initiated at 4.5 L/min with FiO_2_ 1.0. once stabilized on ECMO, the patient was immediately transported to the catheterization laboratory, where pulmonary angiography confirmed massive bilateral pulmonary emboli with nearly complete occlusion of both main pulmonary arteries (Miller Index 24/34). Right heart catheterization showed severely elevated pulmonary pressures (65/32 mm Hg, mean 43 mm Hg). Catheter-directed mechanical thrombectomy with the FlowTriever device was performed with significant thrombus removal (estimated > 70% reduction in thrombus burden). Post-thrombectomy angiography showed marked improvement in pulmonary blood flow with reduction in pulmonary arterial pressures to 38/15 mm Hg (mean 24 mm Hg).

Following the combined intervention, the patient showed rapid hemodynamic improvement with decreasing requirements for vasopressor support. His ECMO flow was gradually weaned as native cardiac output improved, with serial echocardiograms showing progressive recovery of right ventricular function (TAPSE improved from 6–16 mm). He was successfully weaned from ECMO on day 3 and extubated on day 5.

Neurological assessment showed intact higher cortical functions with only mild short-term memory deficits that resolved before discharge. He recovered with excellent neurological outcomes (CPC 1) and was discharged home on day 16 with appropriate anticoagulation therapy (rivaroxaban 15 mg twice daily for 21 days, then 20 mg daily).

### 2.6. Case 6: combined therapy (MCS + ET)

A 45-year-old female with obesity (BMI 34.2 kg/m²) and hormonal contraceptive use presented with progressive dyspnea and right leg pain over 2 days. Wells score was 8, indicating high PE probability. In the emergency department, she developed CA with PEA. After 10 minutes of conventional CPR without ROSC, bedside echocardiography showed severe right ventricular dilation with RV/LV ratio > 1.6, severe tricuspid regurgitation, and estimated RVSP of 68 mm Hg.

The PE-CA protocol was activated with rapid deployment of VA-ECMO. ECMO flow was established at 4.0 L/min, achieving circulatory support at 12 minutes from arrest time. Initial blood gases on ECMO showed severe metabolic acidosis (pH 7.08, lactate 15.2 mmol/L, base excess −16) that gradually improved with ECMO support.

She was immediately transferred to the interventional suite where pulmonary angiography confirmed massive bilateral pulmonary emboli (Miller Index 22/34) with severely reduced pulmonary perfusion. She underwent catheter-directed suction thrombectomy with the Indigo system and catheter-directed thrombolysis with alteplase (24 mg total, 12 mg in each pulmonary artery, delivered directly into pulmonary arteries). Post-intervention angiography demonstrated significant improvement in pulmonary perfusion with reduction in pulmonary arterial pressure from 58/30 mm Hg to 32/14 mm Hg.

The patient showed remarkable improvement after combined therapy, allowing for rapid weaning of vasopressor support within 24 hours. Serial echocardiograms showed progressive improvement in right ventricular function (TAPSE improved from 5–14 mm on day 1 and 19 mm on day 4). She was successfully decannulated from ECMO on day 2, and extubated on day 3.

She recovered with excellent neurological function (CPC 1) without cognitive deficits or focal neurological signs. She was discharged on day 11 on appropriate anticoagulation (enoxaparin bridging to warfarin).

### 2.7. Case 7: combined therapy (MCS + ET)

A 41-year-old male patient presented with lower limb pain and edema 3 months ago, as well as a recent history of consuming a large amount of carbonated beverages 10 days prior (Wells score 7.5, high PE probability). He was brought back to our hospital for emergency treatment due to acute respiratory distress and chest pain. Within minutes of arrival at the intensive care unit, he developed CA with PEA. After initiating standard CPR, point-of-care echocardiography revealed acute right ventricular failure with significant dilation (RV/LV ratio > 1.7) and minimal LV filling.

Given the high clinical suspicion for PE-CA, the rapid-response ECMO team was activated while CPR continued. ECMO was initiated at 4.3 L/min flow rate with FiO_2_ 1.0, establishing circulatory support at 12 minutes from arrest onset. Initial laboratory studies showed severely elevated D-dimer (23.17 μg/mL), troponin I (1.6 ng/mL), and lactate (9.3 mmol/L).

Once stabilized on ECMO support, the patient was transferred to the catheterization laboratory for emergency pulmonary angiography, which confirmed massive bilateral pulmonary emboli with nearly complete occlusion of the right main pulmonary artery and significant thrombus burden in the left pulmonary arterial system (Miller Index 26/34). He underwent rheolytic thrombectomy using the AngioJet system with pulse-spray technique and additional manual aspiration thrombectomy, achieving significant thrombus removal (estimated > 65% reduction).

Post-intervention pulmonary angiography showed marked improvement in pulmonary blood flow with reduction in pulmonary arterial pressure from 62/28 mm Hg to 35/15 mm Hg. Hemodynamic parameters improved substantially following the combined intervention, with mean arterial pressure increasing from 55 mm Hg to 78 mm Hg despite reduction in vasopressor doses.

Following combined MCS and ET intervention, the patient showed progressive improvement in right ventricular function on serial echocardiograms (TAPSE improved from 7–12 mm on day 2 and 18 mm on day 6). He was successfully weaned from ECMO on day 3.

He required 8 days of mechanical ventilation but recovered with good neurological outcomes (CPC 2) with mild cognitive impairment that improved with rehabilitation. He was discharged to rehabilitation on day 14 with continued improvement in functional status. The clinical outcomes for each case are detailed in Table 1.

**Table 1:** The clinical outcome of each case.

Parameters	Age	Wells score	NEWS score	RV/LV ratio	D-Dimer (µg/mL)	Treatment	ECMO	ROSC (min)	CPC	LOS (day)	Outcome
Case 1	62	6	7	>1.5	8.7	Alteplase	N/A	28	5	14	Poor
Case 2	71	7.5	6	>1.2	10.2	Urokinase	N/A	32	4	18	Poor
Case 3	58	9	12	N/A	15.6	Low-molecular-weight heparin	Treated	22	2	15	Poor
Case 4	49	8.5	13	N/A	18.2	Endovascular thrombectomy	N/A	18	1	28	Poor
Case 5	52	7	15	>1.8	16.8	Endovascular thrombectomy	Treated	14	1	16	Favorable
Case 6	45	8	12	>1.6	22.0	Endovascular thrombectomy	Treated	12	1	11	Favorable
Case 7	41	7.5	13	N/A	23.17	Endovascular thrombectomy	Treated	10	0	14	Favorable

Outcomes were categorized as follows: favorable outcome (full recovery): survival without significant cognitive or organ dysfunction at discharge. Poor outcome: includes all-cause in-hospital mortality, survival with cognitive impairment, and survival with persistent organ dysfunction.

CPC = cerebral performance category, ECMO = extracorporeal membrane oxygenation, LOS = length of stay, N/A = not applicable, NEWs = national early warning score, ROSC = return of spontaneous circulation, RV/LV ratio = right ventricle to left ventricle ratio.

## 3. Discussion

This case series describes the clinical outcomes of 3 treatment strategies for PE-CA: conventional therapy, single intervention (MCS or ET), and combined therapy (MCS + ET). The 7 cases presented suggest distinct outcome patterns that may inform future research directions.

The cases treated with conventional therapy (Cases 1 and 2) illustrate the challenges associated with standard advanced cardiac life support and systemic thrombolysis in PE-CA. Despite ultimately achieving ROSC, both patients experienced prolonged resuscitation times (28 and 32 minutes), resulting in multiorgan failure and unfavorable neurological outcomes. These observations align with previous reports indicating high mortality rates (65–95%) with conventional management.^[3]^ The hemodynamic disruption caused by massive PE may limit the effectiveness of standard CPR, as chest compressions may be insufficient to overcome obstructed pulmonary vasculature and restore adequate left ventricular filling.^[6]^

Systemic thrombolysis, while beneficial in some high-risk PE scenarios,^[14]^ may not be sufficient in CA settings. In their meta-analysis of PE-CA patients, systemic thrombolysis during CPR showed only modest benefit, with significant heterogeneity in outcomes.^[7]^ The delayed action of thrombolytics (typically 30–60 minutes to achieve significant clot dissolution) may explain why conventional therapy failed to prevent neurological injury in our cases, as prolonged low-flow states persisted despite thrombolytic administration.^[15]^

Patients receiving single interventions (Cases 3 and 4) exhibited improved yet still suboptimal outcomes. In the MCS-only case (Case 3), mechanical support stabilized circulation but did not directly address the underlying thrombus burden, potentially delaying complete recovery. The patient required 7 days of ECMO before native circulation stabilized, which is consistent with prior observations that MCS alone may require extended support until endogenous fibrinolysis resolves pulmonary obstruction.^[9]^

Conversely, the ET-only case (Case 4) showed how endovascular intervention can effectively remove thrombi but may not provide sufficient hemodynamic support during the critical intervention period. The patient experienced recurrent arrest during the procedure, highlighting the precarious hemodynamic state that persists despite ongoing thrombus removal. This observation is aligns with findings from the PERFECT registry, where patients with massive PE undergoing catheter-directed therapies without MCS had a procedural complication rate of 16%, including hemodynamic collapse in 4.3% of cases.^[12]^ Nevertheless, the favorable neurological outcome in this case highlights the importance of timely anatomical intervention.

In the combined therapy cases (Cases 5–7), shorter ROSC times (12–16 minutes), earlier clinical improvement, and favorable neurological outcomes were observed. While these findings are preliminary and based on a small number of cases, they suggest that the combination of MCS and ET may offer a more comprehensive approach to managing the dual pathophysiological challenges of PE-CA. MCS, particularly VA-ECMO, provides immediate circulatory support, preventing end-organ damage and creating a stable platform for intervention, while ET directly treats the underlying cause by removing the obstructing thrombi, catheter-directed therapy directly addresses the anatomical obstruction, reducing right ventricular afterload and facilitating recovery of native cardiac function.^[16]^ This dual approach may address both the hemodynamic instability and the mechanical obstruction more effectively than either modality alone. These observations are consistent with findings from a systematic review that reported improved 30 days survival with combined strategies compared to monotherapy.^[17]^

Neurological outcomes in this series may be associated with the duration and severity of cerebral hypoperfusion. In PE-CA, both the severity and duration of cerebral hypoperfusion directly impact neurological recovery.^[18]^ In our conventional therapy cases, both experienced poor neurological outcomes (death or CPC 4), which may reflect the prolonged low-flow states during resuscitation. The single intervention cases showed moderate neurological recovery (CPC 2) in the MCS-only case and good recovery (CPC 1) in the ET-only case, though both experienced extended periods of hemodynamic instability. In contrast, all 3 combined therapy cases achieved good to excellent neurological outcomes (CPC 1–2), These findings suggest a potential link between this approach and improved cerebral perfusion, possibly due to shorter low-flow duration. However, given the small sample size, this remains exploratory. The earlier implementation of MCS and ET may contribute to these observations, as reflected in the shorter ROSC times in the combined therapy cases. These findings are consistent with recent reports suggesting that delays in initiating mechanical support during refractory CA may increase the risk of poor neurological outcomes%.^[19]^

The technical feasibility of this approach has improved substantially in recent years, with the development of rapid-deployment ECMO systems and streamlined catheter-based thrombectomy techniques.^[20]^ A recent review revealed that centers with multidisciplinary PE response teams are increasingly adopting combined approaches for the highest-risk patients.^[21]^ These cases support the evolving discussion on the role of early combined therapy in PE-CA, although larger studies are needed to define its precise indications and outcomes.^[13]^

This study has several limitations. First, it is a retrospective case series with a small sample size, which limits the ability to perform statistical analysis and draw definitive conclusions. As a result, our findings should be interpreted as hypothesis-generating rather than confirmatory. Second, some clinical data were missing likely due to incomplete documentation at the time of admission. While these parameters may provide additional insights, their absence does not significantly affect the main observations of this case series. Third, there were differences in baseline characteristics such as age across groups, which may influence outcomes. However, due to the limited sample size, we were unable to adjust for potential confounders. Fourth, the small sample size also limited our ability to comprehensively assess device-related complications (e.g., bleeding, limb ischemia, infection) associated with MCS or ECMO use. Fifth, only 1 patient (Case 2) had a significant comorbidity (cancer), and we did not perform a formal analysis of its potential impact on outcome due to the lack of variability in comorbid status across the cohort.

## 4. Conclusion

This case series highlights the potential feasibility of integrating MCS and ET for managing PE-CA, with observations suggesting a possible association between combined therapy and improved survival trends or neurological recovery. The approach may address both hemodynamic instability and thrombotic burden, offering a hypothesis for further investigation as a rescue strategy in high-risk patients. To further validate these observations, additional research involving larger, multicenter patient cohorts is necessary to confirm our findings and refine the selection criteria for this therapeutic approach.

## Author contributions

**Conceptualization:** Yuanjun Zhang, Haojie Xiong, Li Zhang, Hao Yang.

**Data curation:** Ji Luo.

**Investigation:** Zheng Lei.

**Methodology:** Dongju Chen.

**Supervision:** Bo Wang, Hao Yang.

**Validation:** Hao Yang.

**Writing – original draft:** Yuanjun Zhang.

**Writing – review & editing:** Hao Yang.
